# Supplementary material for: Development of a Transformation Method for Metschnikowia borealis and other CUG-Serine Yeasts
Source: Genes (Basel). 2019 Jan 23;10(2):78. doi: 10.3390/genes10020078 (PMC6409616; doi:10.3390/genes10020078)
Supplement: Supplementary file 1 [file genes-10-00078-s001.pdf]

Development of a Transformation Method for Metschnikowia borealis and other CUG-Serine Yeasts

Zachary B. Gordon [1,2], Maximillian P.M. Soltysiak [3], Christopher Leichthammer [1], Jasmine A. Therrien [1], Rebecca S. Meaney [1], Carolyn Lauzon [1], Matthew Adams [1], Dong Kyung Lee [3], Preetam Janakirama [1], Marc-André Lachance [3] and Bogumil J. Karas [1,2,*]

[1] Designer Microbes Inc., London, ON N6G 4X8, Canada; zgordon2@uwo.ca (Z.B.G.); cleichth@uwo.ca (C.L.); jasmine.alyssa.therrien@gmail.com (J.A.T.); rmeaney2@uwo.ca (R.S.M.); carolyn.lauzon@gmail.com (C.L.); adams.mil@hotmail.com (M.A.); preetam.janakirama@gmail.com (P.J.)

[2] Department of Biochemistry, Schulich School of Medicine and Dentistry, University of Western Ontario, London, ON N6A 5C1, Canada

[3] Department of Biology, University of Western Ontario, London, ON N6A 5B7, Canada; msoltys4@uwo.ca (M.P.M.S); dlee335@uwo.ca (D.K.L.); lachance@uwo.ca (M.-A.L.)

* Correspondence: bkaras@uwo.ca

Supplementary Figures and Tables:

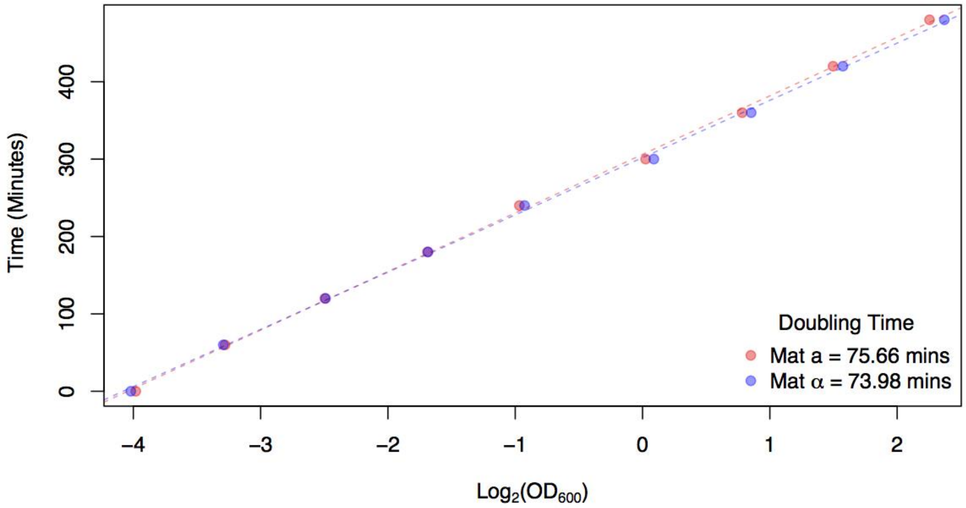

Supplementary Figure S1. Growth rate of *M. borealis MATa* and *MATα*. Three cultures of each mating type of *MATa* and *MATα* were grown to mid-log phase (OD_{600} = 1.0), and diluted in 50 mL of YPAD to an optical density of 0.065. Each culture was then grown at 30°C with shaking at 225 rpm, and OD_{600} was recorded at 1-hr time points for 8 hours. The average OD_{600} of the three cultures for each mating type was recorded at each time point, and the doubling times were calculated as the slopes of the lines of best fit of $Log_2(OD_{600})$ versus each time point in minutes.

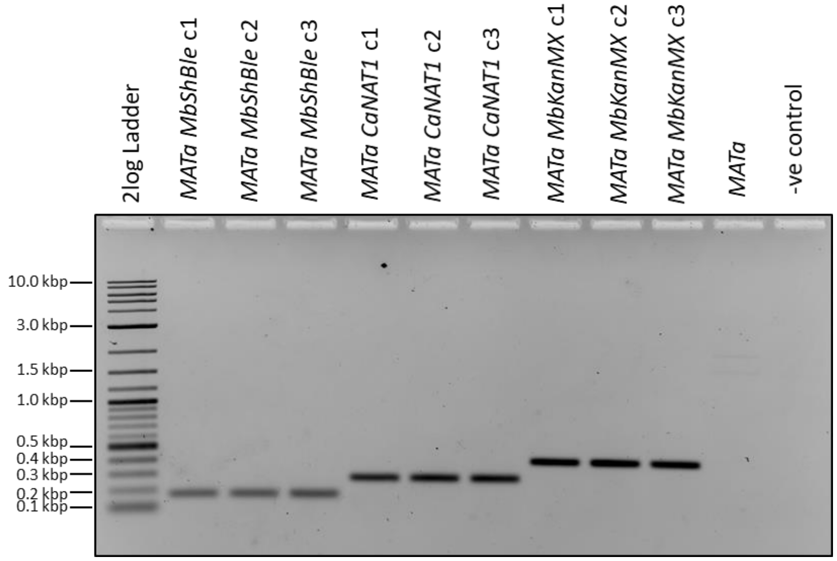

Supplementary Figure S2. Genotyping transformants. Three colonies of *M. borealis MATa* that were transformed with *MbShBle*, *CaNAT1*, and *MbKanMX* (Figure 2) were genotyped by Multiplex PCR (Quiagen) using primers that amplify each selectable marker. Expected sizes were 185 base-pairs (*MbShBle*), 283 base-pairs (*CaNAT1*), and 404 base-pairs (*MbKanMX*). Two negative controls include PCR performed with DNA isolated with untransformed MATa strain as well as without any DNA (-ve control). The PCR was run for 28 cycles with all three sets of primers present in each reaction.

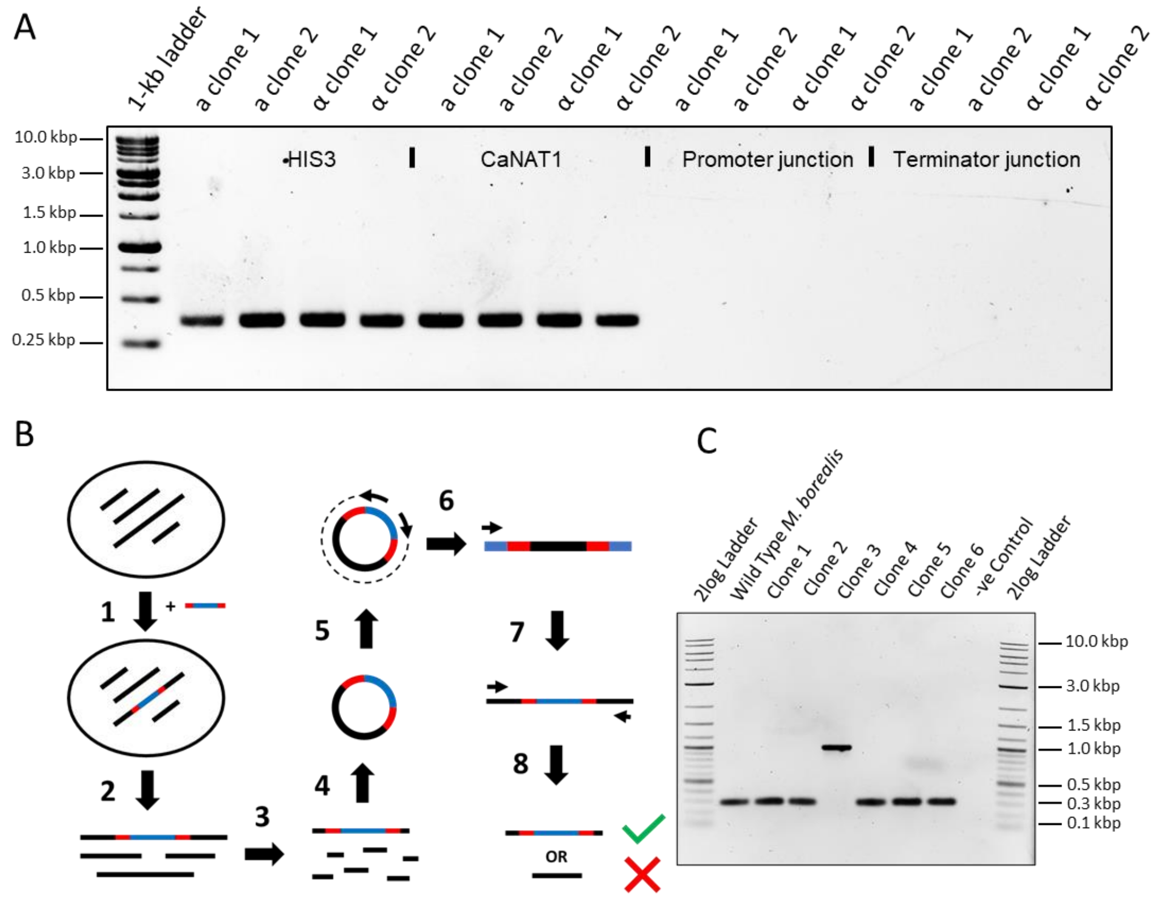

Supplementary Figure S3. Identification of insertion sites. *M. borealis* mating types a and *α* were transformed with a PCR-linearized *CaNAT1* in yeast alternative nuclear code, flanked by 60 base-pair sequences of the *M. borealis HIS3* promoter and terminator by electroporation. (A) Two colonies of a and *α* mating types were screened by PCR to look for a targeted knockout of *HIS3*. Lanes 2-5 used primers that bind within the *M. borealis HIS3* gene (expected size 416 base pairs), lanes 6-9 used primers that bind within the *CaNAT1* marker (expected size 388 base pairs), lanes 10-14 used primers to amplify across the *HIS3* promoter-*CaNAT1* insertion junction (expected size 682 base-pairs), and lanes 14-17 used primers to amplify across the *CaNAT1*-*HIS3* terminator junction (expected size 779 base-pairs). (B) Schematic of the protocol used to identify the insertion site: 1) Lithium acetate/electroporation to transform *M. borealis* with the marker DNA for insertion; 2) Alkaline lysis to isolate *M. borealis* DNA; 3) Restriction digest with CfoI; 4) Ligate sticky ends with T4 ligase; 5) PCR amplify the adjacent genomic DNA; 6) Sequence PCR product; 7) Design primers ~150 base-pairs upstream and downstream of the insertion site; 8) PCR amplify expected insertion site. (C) Confirmation of one insertion site. Primers were designed to amplify across the insertion site identified in clone 3, and the site was PCR-amplified in wildtype *M. borealis*, as well as transformants 1-6. Expected size of the site is ~300 base-pairs (wildtype) and ~1000 base-pairs (with the *CaNAT1* vector insertion).

Supplementary Table S1. Identification of antibiotic sensitivity. Cultures of *M. borealis MATa* and *MAT*α were grown to OD_{600} of 1.5, concentrated to OD_{600} = 3.0, and three dilutions were plated onto YPAD with various concentrations of zeocin or nourseothricin or combination of these two, or geneticin (G418). Plates were incubated for 2-4 days, and colonies were counted. Zeo = zeocin; NTC = nourseothricin; G418 = geneticin; NG = no growth; C = confluent growth.

	MATa						***MATα***					
Growth Time	2 days			4 days			2 days			4 days		
Dilution Factor	10^0	10^1	10^2	10^0	10^1	10^2	10^0	10^1	10^2	10^0	10^1	10^2
Zeo 50 mg L^{-1}	1	NG	NG	7	2	NG	2	NG	NG	8	3	1
Zeo 75 mg L^{-1}	NG	NG	NG	1	NG	NG	NG	NG	NG	1	1	NG
Zeo 100 mg L^{-1}	NG	NG	NG	1	NG	NG	NG	NG	NG	NG	NG	NG
Zeo 125 mg L^{-1}	NG	NG	NG	NG	NG	NG	NG	NG	NG	NG	NG	NG
NTC 50 mg L^{-1}	NG	NG	NG	NG	NG	NG	NG	NG	NG	NG	NG	NG
NTC 75 mg L^{-1}	NG	NG	NG	NG	NG	NG	NG	NG	NG	NG	NG	NG
NTC 100 mg L^{-1}	NG	NG	NG	NG	NG	NG	NG	NG	NG	NG	NG	NG
NTC 125 mg L^{-1}	NG	NG	NG	NG	NG	NG	NG	NG	NG	NG	NG	NG
Zeo 25 mg L^{-1} NTC 25 mg L^{-1}	NG	NG	NG	NG	NG	NG	NG	NG	NG	NG	NG	NG
Zeo 25 mg L^{-1} NTC 50 mg L^{-1}	NG	NG	NG	NG	NG	NG	NG	NG	NG	NG	NG	NG
Zeo 50 mg L^{-1} NTC 25 mg L^{-1}	NG	NG	NG	NG	NG	NG	NG	NG	NG	NG	NG	NG
Zeo 50 mg L^{-1} NTC 50 mg L^{-1}	NG	NG	NG	NG	NG	NG	NG	NG	NG	NG	NG	NG
G418 200 mg L^{-1}	C	C	NG	C	C	C	C	C	NG	C	C	C
G418 300 mg L^{-1}	NG	NG	NG	8	1	NG	NG	NG	NG	12	1	NG
G418 400 mg L^{-1}	NG	NG	NG	2	NG	NG	NG	NG	NG	1	NG	NG

Supplementary Table S2. Transformation efficiencies for *M. borealis*. Cultures of *MATa* and *MATα* were transformed with *CaNAT1* by electroporation and lithium acetate methods alongside negative controls in triplicate, plated on YPAD with 75 mg L^{-1} nourseothricin, and incubated for 48 hrs at 30°C for colonies to appear. No colonies grew on any of the control plates. Efficiency is given as colony forming units per µg of DNA for 10^8 cells. Av = average efficiency for each strain; Electro = electroporation; LiOAc = lithium acetate.

Strain	Method	# Cells	Vector DNA (µg)	Plated	Colonies	Efficiency	Av.
MATa	Electro.	10^8	1	2%	45	2250	
	Electro.	10^8	1	2%	13	650	1600
	Electro.	10^8	1	2%	38	1900	
MATα	Electro.	10^8	1	2%	9	450	
	Electro.	10^8	1	2%	19	950	817
	Electro.	10^8	1	2%	21	1050	
MATa	LiOAc	10^8	1	100%	13	13	
	LiOAc	10^8	1	100%	4	4	9
	LiOAc	10^8	1	100%	9	9	
MATα	LiOAc	10^8	1	100%	5	5	
	LiOAc	10^8	1	100%	1	1	4
	LiOAc	10^8	1	100%	5	5	

Supplementary Table S3. Double marker transformation of *M. borealis*. *M. borealis MATa* was transformed with both the *CaNAT1* and *MbShble* cassettes in the same reaction, and plated on YPAD with 200 mg L^{-1} zeocin, YPAD with 100 mg L^{-1} nourseothricin, and YPAD with 200 mg L^{-1} zeocin and 100 mg L^{-1} nourseothricin. Plates were incubated for 48 hrs at 30°C for colonies to appear. Percentage of double transformants was calculated by dividing the number of colonies on the double selection plate by the average number of colonies on the single selection plates. NTC = plates containing nourseothricin; Zeo = plates containing zeocin; NTC/Zeo = double selection plates containing nourseothricin and zeocin.

Note: In addition to data presented in this table, 200 colonies from the 200 mg L^{-1} zeocin selection plate were re-streaked onto YPAD with 100 mg L^{-1} nourseothricin. From the 200 colonies tested, four colonies were able to grow on plates containing 100 mg L^{-1} nourseothricin.

		Colonies			
**Strain**	**Replicate**	**NTC**	**Zeo**	**NTC/Zeo**	**% Double Transformants**
MATa	1	513	835	19	2.8
	2	514	833	9	1.3
	3	442	707	10	1.7
Average		490	792	13	2.0

Supplementary Table S4. Transformation results for additional yeast strains. An additional 19 yeast strains were transformed by electroporation using the PCR-linearized *CaNAT1* gene (in standard code or yeast alternative nuclear code) flanked by 60-base-pair *ADH1* promoter and terminator sequences from *M. borealis*. Transformants were plated on YPAD with 75-200 mg L^{-1} nourseothricin and incubated at 30°C for 2 days until colonies appeared. *M.* aff *bentonensis, M. bicuspidata,* and *M. orientalis* were incubated at 27°C for 4 days until colonies appeared. NTC = nourseothricin; Alt. = *CaNAT1* in yeast alternative nuclear code; Std. = *CaNAT1* in standard code; Ctrl. = negative control.

Note: Small background colonies began to appear for *M. picinguabensis*, *M. saopaulonensis*, and *M. reukaufii* at lower concentrations of nourseothricin due to natural antibiotic resistance, but background resistance was eliminated when transformations were re-plated on YPAD with 300 mg L^{-1} nourseothricin.

			Number of Colonies		
**Strain**	**NTC (mg L^{-1})**	**Plated**	**Alt.**	**Std.**	**Ctrl.**
Candida aff *bentonensis*	75	40%	27	30	0
Candida bromeliacearum	100	10%	54	0	0
Candida intermedia	100	100%	15	0	0
Candida picinguabensis	100	20%	321	0	0
Candida pseudointermedia	100	20%	47	0	0
Candida saopaulonensis	200	10%	42	0	0
Candida tolerans	100	10%	25	0	0
Candida ubatubensis	100	20%	41	0	0
Clavispora lusitaniae	75	40%	16	0	0
Metschnikowia agaves	100	10%	25	0	0
Metschnikowia bicuspidata	75	40%	4	0	0
Metschnikowia caudate	100	20%	9	0	0
Metschnikowia drosophilae	100	40%	4	0	0
Metschnikowia gelsemii	100	10%	93	0	0
Metschnikowia gruessii	100	10%	37	0	0
Metschnikowia lunata	100	10%	64	0	0
Metschnikowia orientalis	100	100%	13	0	0
Metschnikowia pulcherrima	100	10%	112	0	0
Metschnikowia rancensis	100	10%	27	0	0
Metschnikowia reukaufii	200	10%	121	0	0
Saccharomyces cerevisiae	100	10%	32	29	0

Primers used in this study:

Primers to amplify *CaNAT1* (with *ADH1* promoter and terminator elements)	
BK420F	TCTTTCTTCACTATTCAAACATACATTGAATACAACCAAGCATCAATTAAGAAAAATGTCTACTACTTTGGATGATACTG
BK420R	AGAAATGCAATGAACGATGAACTATTTATTGTGTATTGGGAGGGGGTCAAAGAGTTTATGGACATGGCATAGACATATAC
Primers to amplify *CaNAT1* (with *HIS3* promoter and terminator elements)	
BK398F	ATTATGACTCTTCCCCAATCTTTTCTCACTCATTACCAATACTAACAGATCAACCCCAAAATGTCTACTACTTTGGATGA
BK398R	CTTCGTATCTATGATTCCTACACCGCATATAGTGGTCACTTAAATAATTCTATATGTCGCTTATGGACATGGCATAGACA
Primers to genotype attempted *HIS3* knock-outs – amplifying *HIS3*	
BK402F	CATGCTCTAGCCAAGCACTCGGGCT
BK402R	CTGATTGCCTCCTTTATGGCGATAG
Primers to genotype attempted *HIS3* knock-outs – amplifying *CaNAT1*	
BK364F	TGTTCCAGGTGATGCTGAAG
BK364R	CAACCACAAATGACCAGCAC
Primers to genotype attempted *HIS3* knock-outs – promoter junction	
BK402F	CATGCTCTAGCCAAGCACTCGGGCT
BK364R	CAACCACAAATGACCAGCAC
Primers to genotype attempted *HIS3* knock-outs – terminator junction	
BK364F	TGTTCCAGGTGATGCTGAAG
BK402R	CTGATTGCCTCCTTTATGGCGATAG
Primers to genotype *M. borealis MATα* clones – amplifying insertion sequences	
BK476F	GTGCTGGTCATTTGTGGTTG
BK478R	TCAATGGTGGATCAACTGGA
Primers to genotype *M. borealis MATα* clones – confirming insertion site in gDNA	
BK523F	AGAGCTGGGCCAATAAGGAG
BK523RA	TTGTCACATCAAGTTTCCTTGG
Multiplex genotyping primers for presence of *MbShBle* gene	
BK578_F	TTGCTGGTGCTGTTGAGTTC
BK578_R	CTCAGCGTACAACTCGTCCA
Multiplex genotyping primers for presence of *CaNAT1* gene	
BK579_F	TCCAGTTGATCCACCATTGA
BK579_R	CAACCACAAATGACCAGCAC
Multiplex genotyping primers for presence of *MbKanMX* gene	

BK580_F	GACGTTACCGACGAGATGGT
BK580_R	TCACCGTGGGTAACAACAGA

Gene sequences:

Wild-type *NAT*:

ATGACCACTCTTGACGACACGGCTTACCGGTACCGCACCAGTGTCCCGGGGGACGCCGAGGCCA
TCGAGGCACTGGATGGGTCCTTCACCACCGACACCGTCTTCCGCGTCACCGCCACCGGGGACGG
CTTCACCCTGCGGGAGGTGCCGGTGGACCCGCCCCTGACCAAGGTGTTCCCCGACGACGAATCG
GACGACGAATCGGACGCCGGGGAGGACGGCGACCCGGACTCCCGGACGTTCGTCGCGTACGGG
GACGACGGCGACCTGGCGGGCTTCGTGGTCGTCTCGTACTCCGGCTGGAACCGCCGGCTGACCGT
CGAGGACATCGAGGTCGCCCCGGAGCACCGGGGGCACGGGGTCGGGCGCGCGTTGATGGGGCT
CGCGACGGAGTTCGCCCGCGAGCGGGGCGCCGGGCACCTCTGGCTGGAGGTCACCAACGTCAAC
GCACCGGCGATCCACGCGTACCGGCGGATGGGGTTCACCCTCTGCGGCCTGGACACCGCCCTGT
ACGACGGCACCGCCTCGGACGGCGAGCAGGCGCTCTACATGAGCATGCCCTGCCCCTGA

***CaNAT1*:**

ATGTCTACTACTTTGGATGATACTGCTTATAGATACAGAACTTCTGTTCCAGGTGATGCTGAAGCT
ATTGAAGCTTTGGATGGTTCTTTCACTACCGATACTGTTTTTAGAGTTACTGCTACTGGTGATGGTT
TCACTTTGAGAGAAGTTCCAGTTGATCCACCATTGACTAAGGTTTTCCCAGATGATGAATCCGAT
GATGAATCCGATGCTGGTGAAGATGGTGATCCAGATTCTAGAACTTTCGTTGCTTATGGTGATGA
TGGTGATTTGGCTGGTTTCGTTGTTGTTTCTTATTCTGGTTGGAACAGAAGATTGACTGTTGAAGAT
ATTGAAGTTGCTCCAGAACATAGAGGTCATGGTGTTGGTAGAGCTTTGATGGGTTTGGCTACTGA
ATTCGCCAGAGAAAGAGGTGCTGGTCATTTGTGGTTGGAAGTTACCAATGTTAATGCTCCAGCTA
TTCATGCTTATAGAAGAATGGGTTTCACTTTGTGTGGTTTGGATACTGCTTTATACGATGGTACTGC
TTCCGATGGTGAACAAGCTTTGTATATGTCTATGCCATGTCCATAA

***CaNAT1* with CUG codons:**

ATGTCTACTACTTTGGATGATACTGCTTATAGATACAGAACTTCTGTTCCAGGTGATGCTGAAGCT
ATTGAAGCTCTGGATGGTTCTTTCACTACCGATACTGTTTTTAGAGTTACTGCTACTGGTGATGGTT
TCACTCTGAGAGAAGTTCCAGTTGATCCACCACTGACTAAGGTTTTCCCAGATGATGAATCCGAT
GATGAATCCGATGCTGGTGAAGATGGTGATCCAGATTCTAGAACTTTCGTTGCTTATGGTGATGA
TGGTGATCTGGCTGGTTTCGTTGTTGTTTCTTATTCTGGTTGGAACAGAAGACTGACTGTTGAAGA
TATTGAAGTTGCTCCAGAACATAGAGGTCATGGTGTTGGTAGAGCTTTGATGGGTTTGGCTACTG
AATTCGCCAGAGAAAGAGGTGCTGGTCATTTGTGGCTGGAAGTTACCAATGTTAATGCTCCAGCT
ATTCATGCTTATAGAAGAATGGGTTTCACTTTGTGTGGTCTGGATACTGCTCTGTACGATGGTACT
GCTTCCGATGGTGAACAAGCTTTGTATATGTCTATGCCATGTCCATAA

Wild-type Sh *ble*:

ATGGCCAAGTTGACCAGTGCCGTTCCGGTGCTCACCGCGCGCGACGTCGCCGGAGCGGTCGAGTT
CTGGACCGACCGGCTCGGGTTCTCCCGGGACTTCGTGGAGGACGACTTCGCCGGTGTGGTCCGGG
ACGACGTGACCCTGTTCATCAGCGCGGTCCAGGACCAGGTGGTGCCGGACAACACCCTGGCCTG
GGTGTGGGTGCGCGGCCTGGACGAGCTGTACGCCGAGTGGTCGGAGGTCGTGTCCACGAACTTC
CGGGACGCCTCCGGGCCGGCCATGACCGAGATCGGCGAGCAGCCGTGGGGGCGGGAGTTCGCC
CTGCGCGACCCGGCCGGCAACTGCGTGCACTTCGTGGCCGAGGAGCAGGACTGA

***MbShBle*:**

ATGGCTAAGTTGACCTCTGCTGTTCCAGTTTTGACCGCTAGAGACGTTGCTGGTGCTGTTGAGTTC
TGGACCGACAGATTGGGTTTCTCTAGAGACTTCGTTGAGGACGACTTCGCTGGTGTTGTTAGAGA
CGACGTTACCTTGTTCATTTCTGCTGTTCAAGACCAAGTTGTTCCAGACAACACCTTGGCTTGGGT
TTGGGTTAGAGGTTTGGACGAGTTGTACGCTGAGTGGTCTGAGGTTGTTTCTACCAACTTCAGAGA
CGCTTCTGGTCCAGCTATGACCGAGATTGGTGAGCAACCATGGGGTAGAGAGTTCGCTTTGAGAG
ACCCAGCTGGTAACTGTGTTCACTTCGTTGCTGAGGAGCAAGACTGA

***MbShBle* with CUG codons:**

ATGGCTAAGTTGACCTCTGCTGTTCCAGTTTTGACCGCTAGAGACGTTGCTGGTGCTGTTGAGTTC
TGGACCGACAGATTGGGTTTCTCTAGAGACTTCGTTGAGGACGACTTCGCTGGTGTTGTTAGAGA
CGACGTTACCCTGTTCATTTCTGCTGTTCAAGACCAAGTTGTTCCAGACAACACCCTGGCTTGGGT
TTGGGTTAGAGGTCTGGACGAGCTGTACGCTGAGTGGTCTGAGGTTGTTTCTACCAACTTCAGAG
ACGCTTCTGGTCCAGCTATGACCGAGATTGGTGAGCAACCATGGGGTAGAGAGTTCGCTCTGAG
AGACCCAGCTGGTAACTGTGTTCACTTCGTTGCTGAGGAGCAAGACTGA

Wild-type *KanMX*:

ATGGGTAAGGAAAAGACTCACGTTTCGAGGCCGCGATTAAATTCCAACATGGATGCTGATTTATA
TGGGTATAAATGGGCTCGCGATAATGTCGGGCAATCAGGTGCGACAATCTATCGATTGTATGGGA
AGCCCGATGCGCCAGAGTTGTTTCTGAAACATGGCAAAGGTAGCGTTGCCAATGATGTTACAGAT
GAGATGGTCAGACTAAACTGGCTGACGGAATTTATGCCTCTTCCGACCATCAAGCATTTTATCCG
TACTCCTGATGATGCATGGTTACTCACCACTGCGATCCCCGGCAAAACAGCATTCCAGGTATTAG
AAGAATATCCTGATTCAGGTGAAAATATTGTTGATGCGCTGGCAGTGTTCCTGCGCCGGTTGCATT
CGATTCCTGTTTGTAATTGTCCTTTTAACAGCGATCGCGTATTTCGTCTCGCTCAGGCGCAATCAC
GAATGAATAACGGTTTGGTTGATGCGAGTGATTTTGATGACGAGCGTAATGGCTGGCCTGTTGAA
CAAGTCTGGAAAGAAATGCATAAGCTTTTGCCATTCTCACCGGATTCAGTCGTCACTCATGGTGA
TTTCTCACTTGATAACCTTATTTTTGACGAGGGGAAATTAATAGGTTGTATTGATGTTGGACGAGT
CGGAATCGCAGACCGATACCAGGATCTTGCCATCCTATGGAACTGCCTCGGTGAGTTTTCTCCTTC
ATTACAGAAACGGCTTTTTCAAAAATATGGTATTGATAATCCTGATATGAATAAATTGCAGTTTC
ATTTGATGCTCGATGAGTTTTTCTAA

***MbKanMX*:**

ATGGGTAAGGAGAAGACCCACGTTTCTAGACCAAGATTGAACTCTAACATGGACGCTGACTTGT
ACGGTTACAAGTGGGCTAGAGACAACGTTGGTCAATCTGGTGCTACCATCTACAGATTGTACGGT

AAGCCAGACGCTCCAGAGTTGTTCTTGAAGCACGGTAAGGGTTCTGTTGCTAACGACGTTACCGA
CGAGATGGTTAGATTGAACTGGTTGACCGAGTTCATGCCATTGCCAACCATCAAGCACTTCATCA
GAACCCCAGACGACGCTTGGTTGTTGACCACCGCTATCCCAGGTAAGACCGCTTTCCAAGTTTTG
GAGGAGTACCCAGACTCTGGTGAGAACATCGTTGACGCTTTGGCTGTTTTCTTGAGAAGATTGCA
CTCTATCCCAGTTTGTAACTGTCCATTCAACTCTGACAGAGTTTTCAGATTGGCTCAAGCTCAATC
TAGAATGAACAACGGTTTGGTTGACGCTTCTGACTTCGACGACGAGAGAAACGGTTGGCCAGTTG
AGCAAGTTTGGAAGGAGATGCACAAGTTGTTGCCATTCTCTCCAGACTCTGTTGTTACCCACGGT
GACTTCTCTTTGGACAACTTGATCTTCGACGAGGGTAAGTTGATCGGTTGTATCGACGTTGGTAGA
GTTGGTATCGCTGACAGATACCAAGACTTGGCTATCTTGTGGAACTGTTTGGGTGAGTTCTCTCCA
TCTTTGCAAAAGAGATTGTTCCAAAAGTACGGTATCGACAACCCAGACATGAACAAGTTGCAAT
TCCACTTGATGTTGGACGAGTTCTTCTAA

***MbKanMX* with CUG codons:**

ATGGGTAAGGAGAAGACCCACGTTTCTAGACCAAGATTGAACTCTAACATGGACGCTGACTTGT
ACGGTTACAAGTGGGCTAGAGACAACGTTGGTCAATCTGGTGCTACCATCTACAGATTGTACGGT
AAGCCAGACGCTCCAGAGTTGTTCCTGAAGCACGGTAAGGGTTCTGTTGCTAACGACGTTACCGA
CGAGATGGTTAGATTGAACTGGCTGACCGAGTTCATGCCATTGCCAACCATCAAGCACTTCATCA
GAACCCCAGACGACGCTTGGTTGTTGACCACCGCTATCCCAGGTAAGACCGCTTTCCAAGTTTTG
GAGGAGTACCCAGACTCTGGTGAGAACATCGTTGACGCTCTGGCTGTTTTCCTGAGAAGATTGCA
CTCTATCCCAGTTTGTAACTGTCCATTCAACTCTGACAGAGTTTTCAGATTGGCTCAAGCTCAATC
TAGAATGAACAACGGTTTGGTTGACGCTTCTGACTTCGACGACGAGAGAAACGGTTGGCCAGTTG
AGCAAGTTTGGAAGGAGATGCACAAGTTGTTGCCATTCTCTCCAGACTCTGTTGTTACCCACGGT
GACTTCTCTTTGGACAACTTGATCTTCGACGAGGGTAAGTTGATCGGTTGTATCGACGTTGGTAGA
GTTGGTATCGCTGACAGATACCAAGACTTGGCTATCTTGTGGAACTGTTTGGGTGAGTTCTCTCCA
TCTTTGCAAAAGAGATTGTTCCAAAAGTACGGTATCGACAACCCAGACATGAACAAGTTGCAAT
TCCACTTGATGTTGGACGAGTTCTTCTAA

***M. borealis ADH1* promoter:**

TTGTCATTGTCAGCAAAGTACATTGATCTGTATTCTTCCAAACCCAAGACATTCTTGACGAAAGCT
TGGATTCTTATAACACTAGCACTTCATGCTTCCACTGCATGCTTCTTCACCAGAATCAATGAAAGT
GGAGAGATCCAAGTAATTGAGCACCTCAAAAATGATGAGACGGTGAAATCCATAGGATTCCTTA
CTCCGTGCCATTCAACACCATGGCATAGTATGTTGCATCGTCCTGAAATCGTTGACTCATCATGGT
TTTTGACATGTGAACCACCATTGCATTTGACCTTTGGTAATATCAACAATGTCAAAGAGTACCGA
GACGAACTGGATCGCTTCTTCGACAATCCCGTGGAGTTTGTACGAAACTTGCCGTATGAGTGGCC
CTCACATCTTGTTGTGTTCGAACCAATGGAGTACTTAGTGACACAGGAATTGCCTCAGTACCATG
AATGCCACAGGTATTTCAATAGCTACTTTCATTGGGACTCACGTCGTCAAGGAGATTTGATTGTGT
TCTGCAAAACAATCTGAGGTGTTGCTTCGTGAATAAATTTCACGTGGAGAAGCGACTTATCTAAA
CGCATAAATGTGTCGCGCATTGAAACACGCATCAAATGCGCTCGTCGGCTAATGTCGGAAAGGC
CGCTCTCGCTCTTCTAAATCTTGTAATCTATCGGGAAATAACTGATATCAAATCATGCCACCCGAC
AATTGCAGCAGATCTGAGACCTGCATAATTATGAGTCAAGAAATATCATAAAATGCGTGCATTGT
ACTTAACTTTAAAGTCTACTCTTTCACTAAAAACCTTAGCATCCTCCTCTGCATGAGTATGCGCTT
AAGTGTCGCAACAAAGCCAGAAATCACACCACGCACATAGAAGCAGCAAACATTCGTGACTAT
AAATATGATGCTTCGCCGGACTCCAGCAATTCTTCTTTCTTCACTATTCAAACATACATTGAATAC
AACCAAGCATCAATTAAGAAAA

***M. borealis ADH1* Terminator:**

ACTCTTTGACCCCCTCCCAATACACAATAAATAGTTCATCGTTCATTGCATTTCTTCTCCAACTCTG
CTATGCGTTCTCTCATCACGTTCTGCTGGATCTGGGTGATCAATTGCTCATCATTCAACACGTCAA
AAAGCTTATTTTTCTCTGCCAACGCATCGCTGATGTTCGCTAGTTTCGAATTCAACCTTTTCATTTT
CATATCCTTCTCTGCCAAGGCCTTTGTCAACTCGTTCACCCGGAGGTTGGCATTCTCCAACTTCAT
GGTTTGTTGATTGAGCCGGGTAATAAGCGATTCCGTTTCTTCATGCAAATGCGAATTTTCTTTAAT
AAGCCGTTCGTTATCAGCATGGCCTGCTGATGTTTTTACATATGCTAATTGCTCAAAAGCCTTCAC
GAAGTCTGCGTCTCGCTTCTCTATCTGGTCACGTTTGTTCAACTGGTCTAGTATTGCATTCCACGGC
TCATTCATTTTGGTTAAAAATGTGAGTCTCCAAGCCCCAATATGGTAATTTGGATGTGTGTTTCAA
GTTTCCTATGTGTCAAATCAGGCTCAGAATGACACATTGCAAGTATCTGGGGTCTTAAATTGCGA
GTGCACAGTGCCACGGTTGGCGCGCTTGATAAGCTTACGACAGAGCATATAACCTCAAACCGAA
ATACACCATCCTAGAATATACTGTCCTATAATATTGACCATTTGAAGAGTCAGTTTTGCTTCTTCA
CTGGTCTAAGAATTGCATTGCACTGCTGCACTAATTGTCTTTAACTCACTTAATTGCCTGTGTATTG
ACATCTGCAAACATCTTCCCCTGTTGATTAGCAAAGAAGCAGGTGAACCGATCGTCTCCTTAGAA
TGGCTGAGCCTTCTTCTTCATGGTGGTCTCTGTTATATCGCTCCGAATCTACCAAACAAGCTGATA
TTTCTTCAAAGGCCACTTCCCCTGGGACACTACCTCCTGCCAACTCTAAAAAGGAAACGAATACT
CAAAAGGCCTC

***M. borealis HIS3* promoter:**

TGTCTAGCTCTAACACCTTGATGGCCACAACCTTTTCAGTTTTCTTGTGATACCCTTTGTAGACGAC
ACCGAACTTGCCCTGGCCGATGACCTTTGTTCTTTGGTAAGAAGAAGTACTTAGCATGGTGAAAG
TGGAGGAGTGTTCTCAAGGCCAAGGACCTAGGGGAATTTGAGATCGAGTACTTTCTCTTGCATGG
AAAGAGAGTTCAGAAGAACGCCTTTGGAACTTCTGCCAATGTTTATAAGCGCTTGAAAACTAAGT
ATGGCGTACAGCGATATCCTGCAGAGTTGATCCGATGGATTATGGTGAAGGCCAGCACGTTGGG
CGTCCAAAACAGAGATCGGGTCGTGAAGGAAAGTGTACCAGTATGAAAATTGAAGGCGCGGTA
GCCAGGATCTGCAGCTGAATACGGTGGGTCGCGCCTGAGAAACAAGATGCACCGAATGCGGAG
GAAGCTTCAAGTAGAGAGCAGAAAGCAAAGATGCTAATTTACGACGAAGGGGTTTTCGAAGGGT
TCGGAGCGTTCGATGCGTTTGCCCAGCCAAATTTGCCAACTAATTCGAAAGCCACCATTGTTAAT
CACCAAAAGGTCAGCTGCCTGAATGGTCAGGTGTACATGTGCAGTCAGTAGACAATAGTGGTGG
ACTTTTGGATTTTCAGGCAAAGAAGTGCGAAGTAAGCTATCAAATGACCAGTGTGCCAACACAC
CATGCAAGAAATTCGGCCACAAGCAAAAAGAGTTGAATTTTCGCCGGCGATGCAAATTAAAAAA
AAAAAAAAAAAGACCCAAAAAACTTAGCCTTCTGTTCCAAAATCAGAAATTGAAAGGTCAGTCT
TTTGTTTGCTCGAATCGGGTACTTCCGAGTTTGCTGCTGCACTGGTCGGGCCATTTTGGTGTGGATG
GAACTTTGGTGAACGGAATTTGCCAGAGATATGCGCATTATGACTCTTCCCCAATCTTTTCTCACT
CATTACCAATACTAACAGATCAACCCCAAA

***M. borealis HIS3* terminator:**

GCGACATATAGAATTATTTAAGTGACCACTATATGCGGTGTAGGAATCATAGATACGAAGCGAA
AAGTCAGAGGTGCGCATTACAACTTTCGGGGCACCGCTTTCGATGCGCATCTCTTCACTACTACC
ATGAGAAGGTTCCACACTAGTGGTATCCGCCAAGTCATCAAGCCAGTGTTCAAACTGCATGATCT
GAAGAAAGGACTCAAGAAATTTGAAGATTCCTTCAATGCAGGTAGTAACCGGAAGCTGGAGCA
GAAAATATGGGACAAGTTGAATATCTCCAAGCACGAGTTTTTCATACGGAAATATGGCAACATTT
CGCCCGAAAAACGAAAACAGTTGGATGACAAAGTCACCCGACAAAGGTCGCTCCGCGAGCAGA
GAAGGAAGAACGAAATGGGCGACGACTACGAATCTTACAGGAAGCCCCGGGCGGCGTTGAACC
CTCTTGCTGAGTACCTCTTTGGCACTCACGCAGTCATGTCCGCATTGACTGCAGGTAAGCGAGAG

GCCTTCAGCACCCTCTACATCCAAAAGTCTAAGGACAGTGTGCGTCAGGTTCTCCTGCTTGCGAA
GAAATATGGAGTCCGGGTCGTGGAGAAGGGATCTAAAGGTGAGATGAACACCTTAAGCTCCAAC
GGTGTCCACAATGGCGTTGTCTTGGAAACCAAGCCCTTGCGCATTCCAGAGGTGTACGAACTTGA
CAAAACTCATGACGGAACCGAAGGACTGTACAATGTTAAAGTGTACGATGAGGAGACGGATTCG
CCAGTGCTGAAAACGTGCCATGTGGCGAGAACAACGGCTGCAAATGCTAACAAATACCCCCTAG
GCATATTTGTGGATGGAATCACCGACCCCCAGAATTTGGGCAATATTATCCGGTCGGCATATTTC
CTAGGTGCAGATTTCCTTGTGATTCCCAATGCAGAGTCTGCTCGTCTAGGCCCCGTTGCTGCCAAA
GCATCAGCCGGCGCGCTTGACCTCATGCCCATAT
